# Lovastatin Treatment Inducing Apoptosis in Human Pancreatic Cancer Cells by Inhibiting Cholesterol Rafts in Plasma Membrane and Mitochondria

**DOI:** 10.3390/ijms242316814

**Published:** 2023-11-27

**Authors:** Momoko Gyoten, Yi Luo, Rina Fujiwara-Tani, Shiori Mori, Ruiko Ogata, Shingo Kishi, Hiroki Kuniyasu

**Affiliations:** 1 Department of Molecular Pathology, Nara Medical University, 840 Shijo-cho, Kashihara 634-8521, Nara, Japan; dc119038@naramed-u.ac.jp (M.G.); lynantong@hotmail.com (Y.L.); m.0310.s.h5@gmail.com (S.M.); pkuma.og824@gmail.com (R.O.); nmu6429@yahoo.co.jp (S.K.); 2 Jiangsu Province Key Laboratory of Neuroregeneration, Nantong University, 19 Qixiu Road, Nantong 226001, China; 3 Research Institute, Nozaki Tokushukai Hospital, 2-10-50 Tanigawa, Daito 574-0074, Osaka, Japan

**Keywords:** statin, pancreas cancer, drug resistance, mitochondrial raft, apoptosis

## Abstract

Resistance to anticancer drugs is a problem in the treatment of pancreatic ductal carcinoma (PDAC) and overcoming it is an important issue. Recently, it has been reported that statins induce apoptosis in cancer cells but the mechanism has not been completely elucidated. We investigated the antitumor mechanisms of statins against PDAC and their impact on resistance to gemcitabine (GEM). Lovastatin (LOVA) increased mitochondrial oxidative stress in PDAC cells, leading to apoptosis. LOVA reduced lipid rafts in the plasma membrane and mitochondria, suppressed the activation of epithelial growth factor receptor (EGFR) and AKT in plasma membrane rafts, and reduced B-cell lymphoma 2 (BCL2)-Bcl-2-associated X protein (BAX) binding and the translocation of F1F0 ATPase in mitochondrial rafts. In the three GEM-resistant cell lines derived from MIA and PANC1, the lipid rafts in the cell membrane and the mitochondria were increased to activate EGFR and AKT and to increase BCL2-BAX binding, which suppressed apoptosis. LOVA abrogated these anti-apoptotic effects by reducing the rafts in the resistant cells. By treating the resistant cells with LOVA, GEM sensitivity improved to the level of the parental cells. Therefore, cholesterol rafts contribute to drug resistance in PDAC. Further clinical research is warranted on overcoming anticancer drug resistance by statin-mediated intracellular cholesterol regulation.

## 1. Introduction

Pancreatic cancer is the third leading cause of cancer-related deaths in the United States [1] and the fourth in Japan [2]. The incidence of pancreatic cancer continues to increase; it is predicted to become the second leading cause of cancer-related deaths in the next decade [3]. Pancreatic ductal adenocarcinoma (PDAC) accounts for the majority of pancreatic cancers; even with multidisciplinary treatment, the prognosis remains poor, with an overall 5-year survival rate of only 8.5% [2]. The early detection of PDAC remains challenging and radical resection is often difficult to perform due to anatomical factors [1]. Consequently, the importance of anticancer drug treatment is increasing. However, the increased likelihood of anticancer drug resistance makes treatment difficult [4].

In terms of chemotherapy for PDAC, improved survival rates have been observed with the use of gemcitabine (GEM), 5-fluorouracil (5-FU), nab-paclitaxel, and folfirinox [5]; however, anticancer drug resistance poses a significant challenge. Resistance to gemcitabine, the primary first-line drug, develops several weeks after treatment initiation [4]. Drug resistance genes and microRNAs [6], the stemness of cancer cells [7], the abundant stroma interacting with cancer cells, and the cancer microenvironment, including the occurrence of hypoxia, are the primary factors that contribute to drug resistance [8]. These factors are difficult to overcome [9]. When PDAC-resistant cell lines induced by continuous treatment with GEM were examined, although the development of specific resistant genes was not triggered, resistance to GEM, 5-FU, cisplatin, and paclitaxel occurred [10,11]. In these resistant cells, the energy metabolism was reprogrammed from oxidative phosphorylation to glycolysis upon treatment with anticancer drugs. As a result, oxidative stress associated with anticancer drug treatment was suppressed. Thus, altered energy metabolism contributes to the acquisition of anticancer drug resistance in PDAC [10,11].

Statin use improves hyperlipidemia, especially hypercholesterolemia, and plays a major role in the prevention of arteriosclerosis and cardiovascular diseases [12,13]. Statins inhibit the rate-limiting reaction of cholesterol synthesis in which 3-hydroxy-3-methylglutaryl coenzyme A (HMGC) is converted to mevalonate by HMGC reductase [14]. As a result, intracellular cholesterol levels decrease. Attempts to use this approach in cancer treatment are attracting attention [15,16]. However, the mechanisms underlying the antitumor effects of statins have not yet been definitively determined.

In this study, we examined the effects of statins on cholesterol rafts by limiting cholesterol production. Lipid rafts are heterogeneous membrane domains characterized by high concentrations of cholesterol, sphingolipids, and gangliosides that serve as sorting platforms to compartmentalize and regulate signal transduction pathways [17]. Cholesterol is essential for lipid raft production [18]. However, for lovastatin (LOVA) and simvastatin (SIMVA), beta-hydroxylation through liver passage is thought to be important for this effect [19,20]. The HMGCoA reductase inhibitory effect of statins relies on the fact that β-hydroxy-oxidized statins are misrecognized as β-hydroxy β-methylglutaryl-CoA, the natural ligand for HMGCoA reductase [19]. Lovastatin and its activated form can reduce cholesterol content through mechanisms beyond enzymatic interaction, influencing the transcription of genes crucial for cholesterol production, metabolism, uptake, and release [19]. We have previously reported that the inhibition of raft production by cholesterol depletion suppresses EGFR signaling and has antitumor effects [21]. Statins reduce the risk of pancreatic cancer and improve the prognosis [22,23,24]. Furthermore, statins induce apoptosis in PDAC cells [25]. However, the mechanism by which statins induce apoptosis by inhibiting cholesterol synthesis has not been completely clarified. In this study, we aimed to investigate the effects of statins on drug resistance in PDAC.

## 2. Results

### 2.1. Antitumor Effects of Statins against PDAC Cells

**Figure 1:**
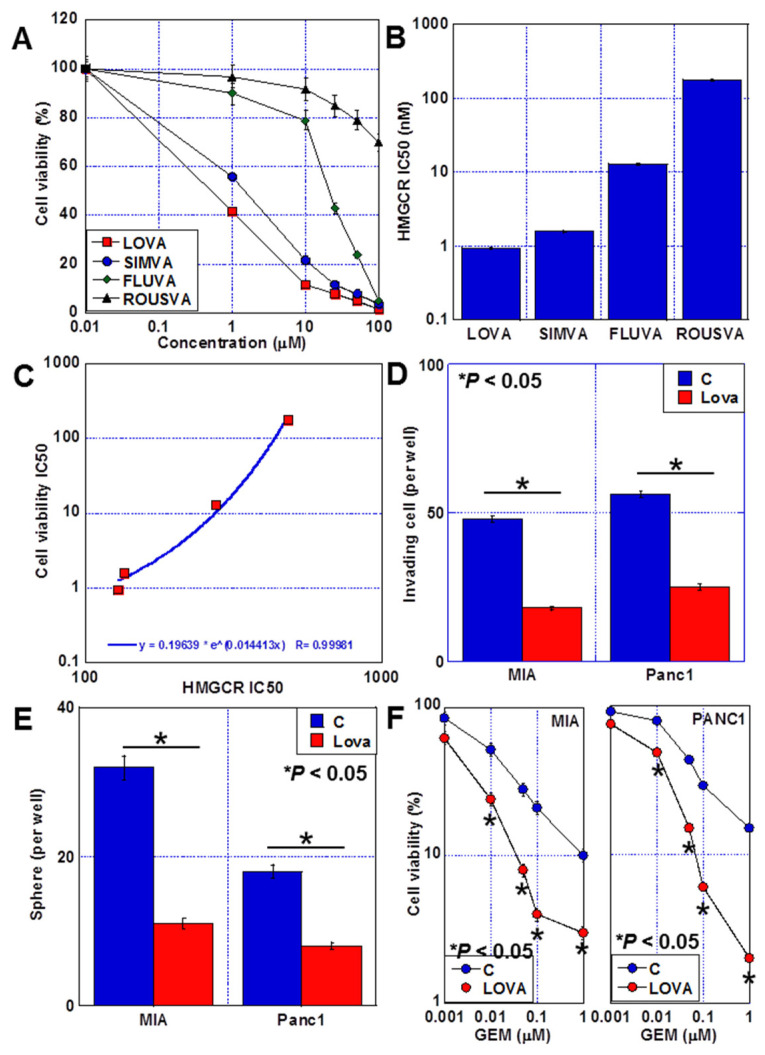
Effect of statins on PDAC cells. (**A**) Effects of statins on PDAC cell proliferation. (**B**) The inhibitory effect of statins on HMG-CoA reductase activity. (**C**) Correlation between HMG-CoA reductase inhibitory activity and growth inhibition by statins. (**D**–**F**) Effects of LOVA on invasion (**D**), sphere formation (**E**), and GEM sensitivity (**F**). Error bars: standard deviation from three independent trials. Statistical significance was calculated using the Mann–Whitney U test. PDAC, pancreatic ductal adenocarcinoma; MIA, MIA-PaCa-2; C, control; LOVA, lovastatin; SIMVA, simvastatin; FLUVA, fluvastatin; ROUSVA, rosuvastatin; HMGCR, 3-hydroxy-3-methylglutaryl coenzyme A reductase; GEM, gemcitabine.

The human PDAC cell lines MIA-PaCa-2 (MIA) and PANC1 were treated with four types of statins (Figure 1A). The antiproliferative effects of LOVA and SIMVA were relatively high, with LOVA being slightly more potent. By contrast, pravastatin (PRAVA) and rosuvastatin (ROUSVA) had poor growth-inhibiting effects. All four statins were ranked as follows based on the potency of their HMG-CoA reductase inhibitory activity in MIA cells: LOVA > SIMVA > PRAVA > ROUSVA (Figure 1B). A correlation was observed between the antiproliferative effect of statins and the inhibitory activity of HMG-CoA reductase (Figure 1C). Furthermore, LOVA, which had the highest antiproliferative effect among the four statins, suppressed the invasive ability and sphere formation of both MIA and PANC1 cells and promoted GEM sensitivity (Figure 1D–F).

### 2.2. Cell Death Caused by LOVA

**Figure 2:**
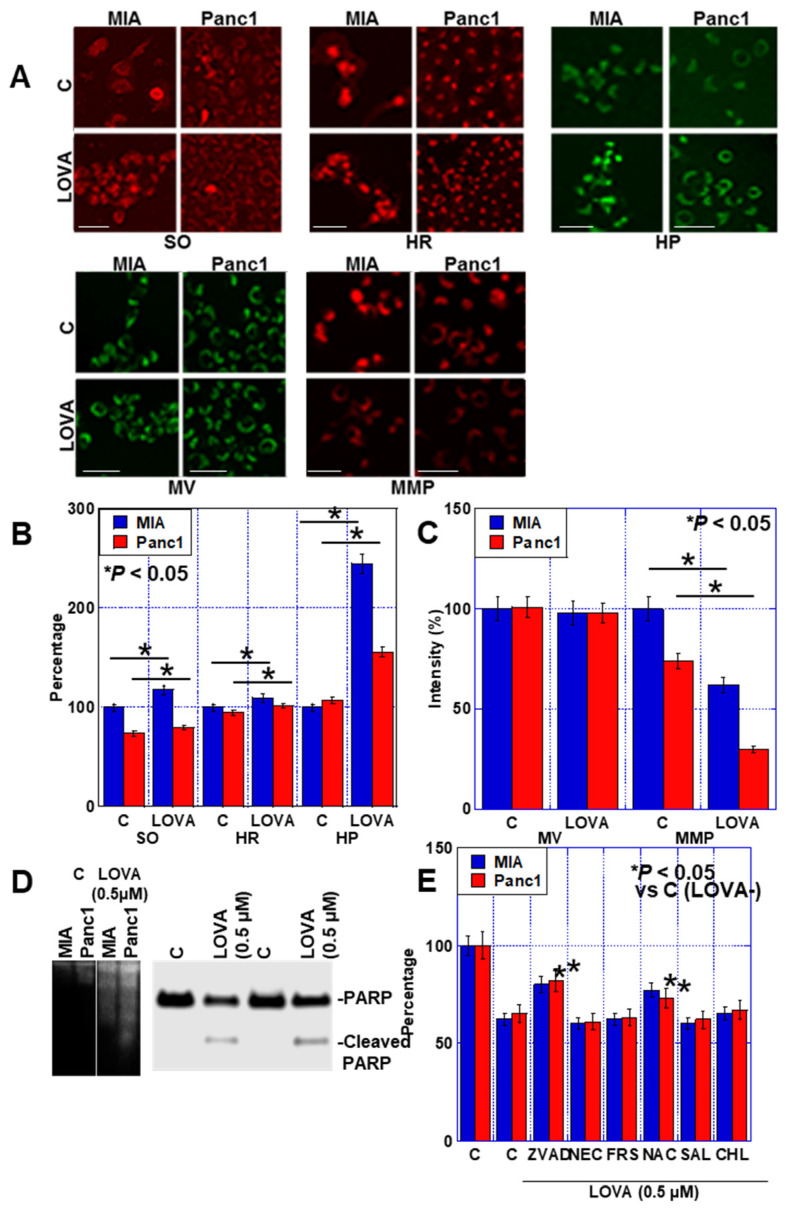
Effect of LOVA on induction of apoptosis. (**A**–**C**) Effect of LOVA on mitochondrial oxidative stress (**A**,**B**), mitochondrial volume, and membrane potential (**A**,**C**). Scale bar, 50 μm. (**D**) DNA ladder formation (left) and PARP cleavage by LOVA. (**E**) Inhibition of cell death. Error bars: standard deviation from three independent trials. Statistical significance was calculated using the Mann–Whitney U test. PDAC, pancreatic ductal adenocarcinoma; MIA, MIA-PaCa-2; C, control; LOVA, lovastatin; SO, superoxide; HR, hydroxy radical; HP, hydrogen peroxide; MV, mitochondrial volume; MMP, mitochondrial membrane potential; PARP, poly (ADP-ribose) polymerase; ZVAD, Z-VAD-FMK; NEC, necrostatin; FRS, ferrostatin; NAC, N-acetyl-L-cysteine; SAL, salubrinal; CHL, chloroquine.

When examining for alterations in mitochondrial oxidative stress due to LOVA, increased levels of superoxide, hydroxyl radicals, and hydrogen peroxide were observed in both PDAC cell lines (Figure 2A,B). LOVA did not reduce the mitochondrial volume but decreased the mitochondrial membrane potential (Figure 2A,C). When LOVA-induced cell death was examined, the ladder formation of genomic DNA and PARP cleavage due to caspase 3 activation were observed, indicating the induction of apoptosis (Figure 2D). During the cell death inhibitor assay, a rescue effect of approximately 20% was achieved following the use of an apoptosis inhibitor (Z-VAD-FMK, ZVAD) and of approximately 15% for N-acetylcystein (NAC) (Figure 2E). However, the use of inhibitors for necroptosis (necrostatin, NEC), ferroptosis (ferrostatin, FRS), autophagy (chloroquine, CHL), and endoplasmic reticulum stress (salubrinal, SAL) did not prevent LOVA-induced cell death. These results suggested that LOVA-induced cell death involves apoptosis due to increased oxidative stress.

### 2.3. Inhibition of Plasma Membrane Rafts by LOVA Treatment

**Figure 3:**
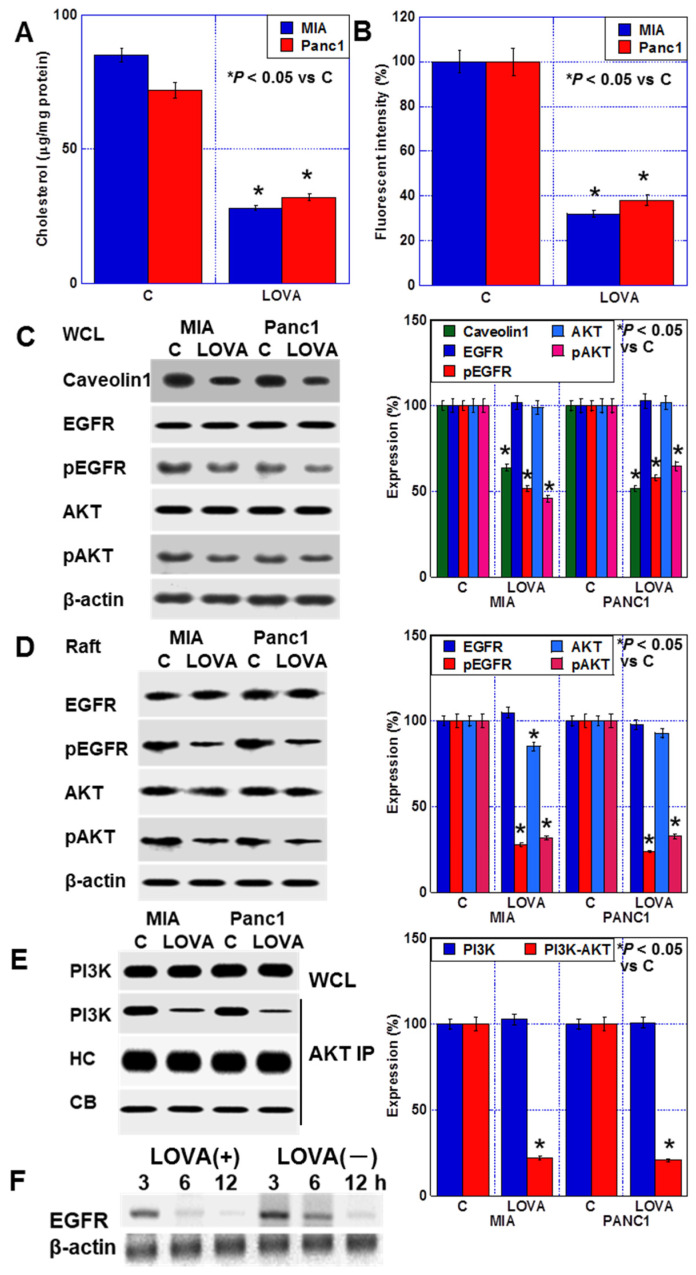
Effect of LOVA on plasma membrane raft. (**A**) Plasma membrane cholesterol content. (**B**) Lipid raft content of the plasma membrane. (**C**,**D**) Effect of LOVA on the activation of EGFR and AKT in whole-cell lysates (**C**) and plasma membrane raft fractions (**D**) (Right panels) Semiquantification of Western blot. (**E**) The effect of LOVA on the interaction between PI3K and AKT in plasma membrane rafts. (Right panel) Semiquantification of Western blot. (**F**) Effect of LOVA on turnover of membranous EGFR. Error bars: standard deviation from three independent trials. Statistical significance was calculated using the Mann–Whitney U test. PDAC, pancreatic ductal adenocarcinoma; MIA, MIA-PaCa-2; C, control; LOVA, lovastatin; EGFR, epithelial growth factor receptor; pEGFR, phosphorylated EGFR; pAKT, phosphorylated AKT; PI3K, phosphoinositide 3-kinases; WCL, whole-cell lysate; IP, immunoprecipitation; HC, heavy chain; CB, Coomassie blue.

To examine the inhibitory effect of LOVA treatment on cholesterol production, we measured the cholesterol content in the membrane fraction of LOVA-treated PDAC cells. The membranous cholesterol content decreased in both cell types (Figure 3A). The semiquantification of cholesterol rafts in the cell membrane using a fluorescent probe using cholera toxin revealed that the rafts were reduced in both cells by LOVA (Figure 3B). In LOVA-treated PDAC cells, the raft-related proteins, such as caveolin-1, epithelial growth factor receptor (EGFR), and AKT [26,27,28] (Figure 3C,D) were detected. Caveolin-1 protein levels were decreased by LOVA in whole-cell lysates. The total protein levels of EGFR and AKT remained unchanged; however, the phosphorylation levels of EGFR and AKT were decreased. Furthermore, the protein levels of EGFR and AKT decreased in the membrane raft fraction, and their phosphorylation levels were lower than those in the whole-cell lysate. Additionally, the physical association between phosphatidylinositol-3 kinase (PI3K) and AKT was reduced in the raft fraction (Figure 3E). Examining the temporal changes in EGFR in the membrane fraction, LOVA promoted the disappearance of EGFR from the membrane (Figure 3F).

### 2.4. Inhibition of Mitochondrial Rafts by LOVA Treatment

**Figure 4:**
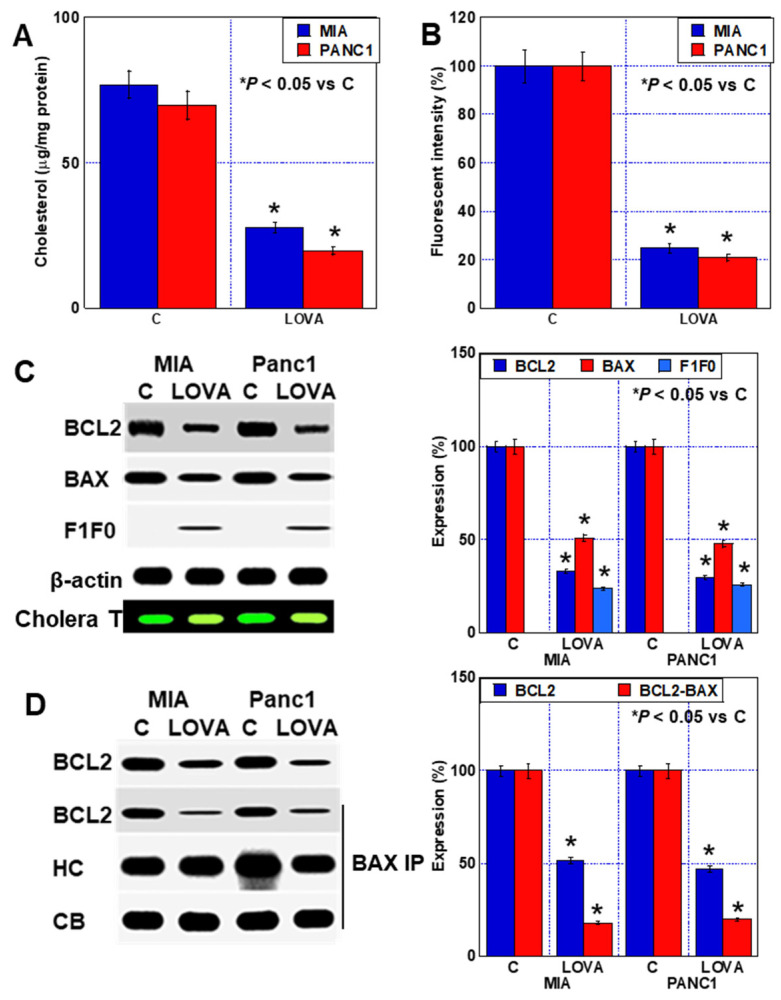
Effect of LOVA on mitochondrial raft. (**A**) Cholesterol content in mitochondria. (**B**) Lipid raft content in the mitochondria. (**C**,**D**) Effect of LOVA on mitochondrial raft proteins (**C**) and BCL2-BAX binding in mitochondrial rafts. (Right panels) Semiquantification of Western blot. (**D**) Error bars: standard deviation from three independent trials. Statistical significance was calculated using the Mann–Whitney U test. PDAC, pancreatic ductal adenocarcinoma; MIA, MIA-PaCa-2; C, control; LOVA, lovastatin; BCL2, B-cell lymphoma 2; BAX, Bcl-2-associated X protein; F1F0, F1F0 ATP synthase subunit C; cholera T, cholera toxin subunit B CF^®^Dye; IP, immunoprecipitation; HC, heavy chain; CB, Coomassie blue.

Thus, the inhibition of cholesterol production by LOVA treatment reduces the number of cholesterol rafts in the cell membrane and suppresses signal transduction. Next, we examined the effects of LOVA treatment on the mitochondrial rafts. When the mitochondria were isolated from the cells and the cholesterol content of the extract was measured, the results showed that the cholesterol content reduced after LOVA treatment (Figure 4A). The semiquantification of mitochondrial cholesterol rafts isolated from LOVA-treated cells using a fluorescent probe revealed that the number of rafts decreased to a lesser extent than that of the plasma membrane rafts (Figure 4B). The raft fractions were extracted from isolated mitochondria and the proteins were examined. The B-cell lymphoma 2 (BCL2) and Bcl-2-associated X protein (BAX) levels were decreased by LOVA treatment (Figure 4C). To confirm that mitochondrial rafts were extracted, the extracts were slot-blotted and stained with cholera toxin. Although the same amount of protein was blotted, cholera toxin binding was reduced by LOVA. The physical association between the two factors also decreased (Figure 4D). Furthermore, F1F0 ATP synthase subunit C was translocated to the mitochondrial rafts following LOVA treatment.

### 2.5. Effect of Cholesterol Adsorbent on PDA Cells

**Figure 5:**
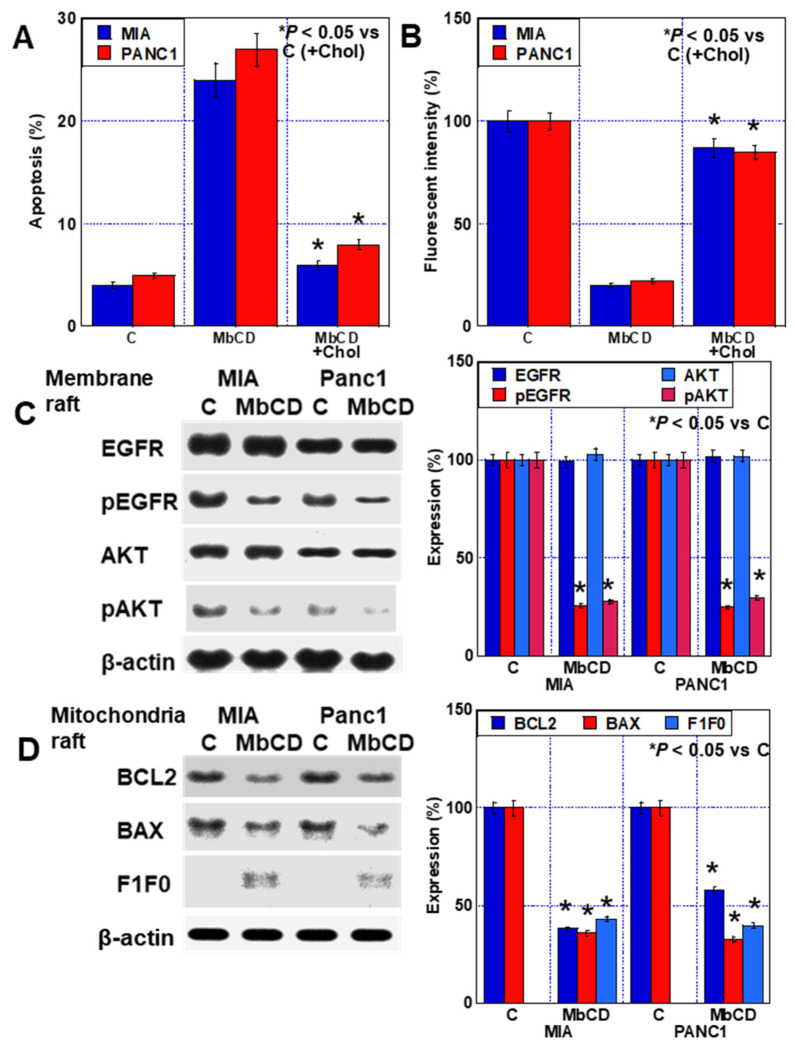
Effect of MβCD on PDAC cells. (**A**) Apoptosis (**B**) Lipid raft content of the plasma membrane. (**C**) Effect of LOVA on the activation of EGFR and AKT in plasma membrane rafts. (Right panel) Semiquantification of Western blot. (**D**) Effect of LOVA on mitochondrial raft proteins. Error bars: standard deviation from three independent trials. (Right panel) Semiquantification of Western blot. Statistical significance was calculated using the Mann–Whitney U test. PDAC, pancreatic ductal adenocarcinoma; MIA, MIA-PaCa-2; C, control; LOVA, lovastatin; MbCD, methyl-β-cyclodextrin; EGFR, epithelial growth factor receptor; pEGFR, phosphorylated EGFR; pAKT, phosphorylated AKT; PI3K, phosphoinositide 3-kinases; BCL2, B-cell lymphoma 2; BAX, Bcl-2-associated X protein; F1F0, F1F0 ATP synthase subunit C.

To confirm whether the antitumor effect of statins lies in the induction of intracellular cholesterol depletion, we investigated the effects of the cholesterol adsorbent methyl-β-cyclodextrin (MβCD) [21,29]. Treatment of PDAC cells with MβCD induced the occurrence of apoptosis and reduced the formation of plasma membrane rafts (Figure 5A,B). In contrast, co-treatment of MβCD with cholesterol abrogated the induction of apoptosis and the reduction of rafts by MβCD. MβCD treatment decreased the phosphorylation levels of EGFR and AKT in plasma membrane rafts (Figure 5B,C). The BCL2 and BAX levels in the mitochondrial rafts were decreased, and F1F0 ATP synthase subunit C was translocated to the rafts (Figure 5D). Thus, treatment with cholesterol adsorbent produced similar alterations in PDAC cells as that of LOVA.

### 2.6. Effect of LOVA on GEM-Resistant PDAC Cells

Three GEM-resistant cell lines were used to evaluate the effects of LOVA treatment on GEM sensitivity. Panc1-G, a GEM-resistant cell line of Panc1, was established by continuous treatment with low concentrations of GEM. MIA-A, which suppresses oxidative stress production, and MIA-B cells, which possess increased stemness, were derived from MIA cells through continuous treatment with low concentrations of GEM in combination with cobalt chloride, an HIF1α inhibitor [11].

**Figure 6:**
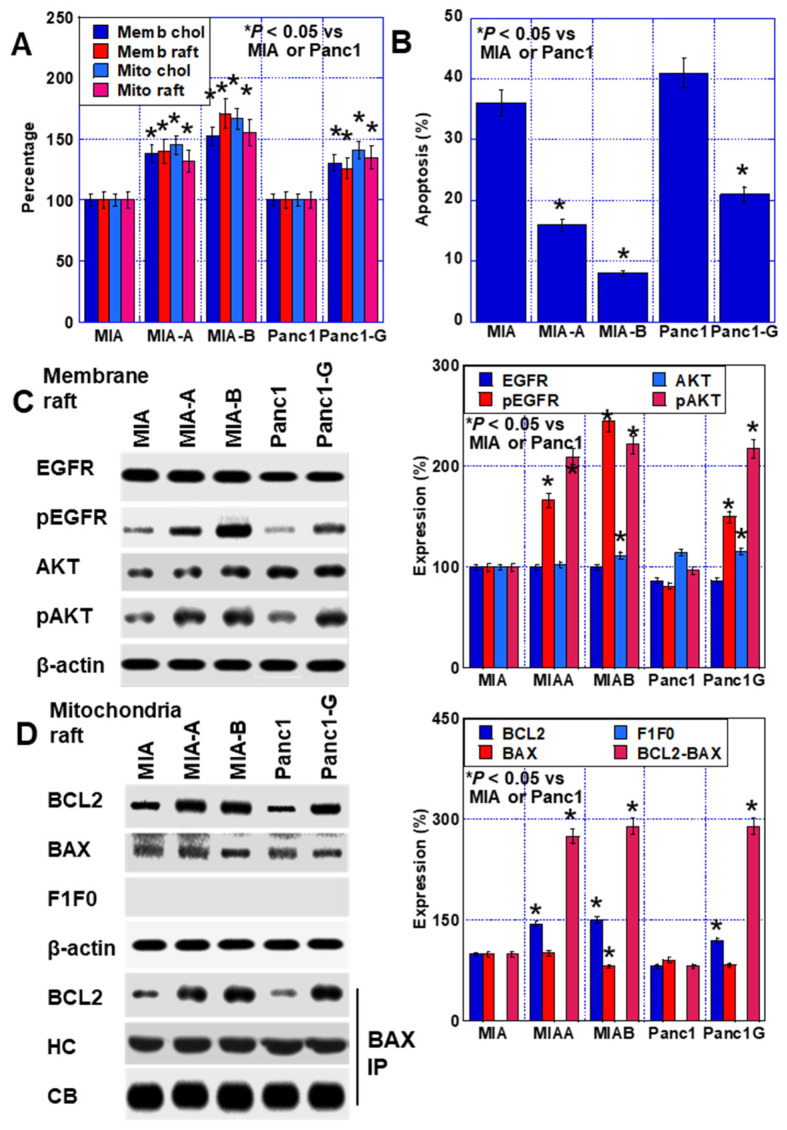
Expression of raft-associated proteins in human GEM-resistant PDAC cells. (**A**) Cholesterol and raft content in the plasma membrane and mitochondria. (**B**) Thapsigargin-induced apoptosis. (**C**) Activation of EGFR and AKT in the plasma membrane raft fraction of GEM-resistant PDAC cells. (Right panel) Semiquantification of Western blot. (**D**) Raft proteins in the mitochondria and BCL2-BAX binding in mitochondrial rafts. (Right panel) Semiquantification of Western blot. Statistical significance was calculated using the Mann–Whitney U test. PDAC, pancreatic ductal adenocarcinoma; MIA, MIA-PaCa-2; C, control; LOVA, lovastatin; EGFR, epithelial growth factor receptor; pEGFR, phosphorylated EGFR; pAKT, phosphorylated AKT; PI3K, phosphoinositide 3-kinases; BCL2, B-cell lymphoma 2; BAX, Bcl-2-associated X protein; F1F0, F1F0 ATP synthase subunit C; GEM, gemcitabine.

In GEM-resistant cells, the cholesterol content in the plasma membrane and the mitochondria was higher than that in parent cells (Figure 6A). The number of rafts also increased. Moreover, the rate of thapsigargin-induced apoptosis in the resistant cells was reduced compared with that in the parent cells (Figure 6B). The phosphorylation levels of EGFR and AKT in the plasma membrane raft fraction increased in the resistant cells (Figure 6C). Furthermore, the binding of BCL2 and BAX in the mitochondrial fraction increased in the resistant cells compared with that in the parent cells (Figure 6D).

**Figure 7:**
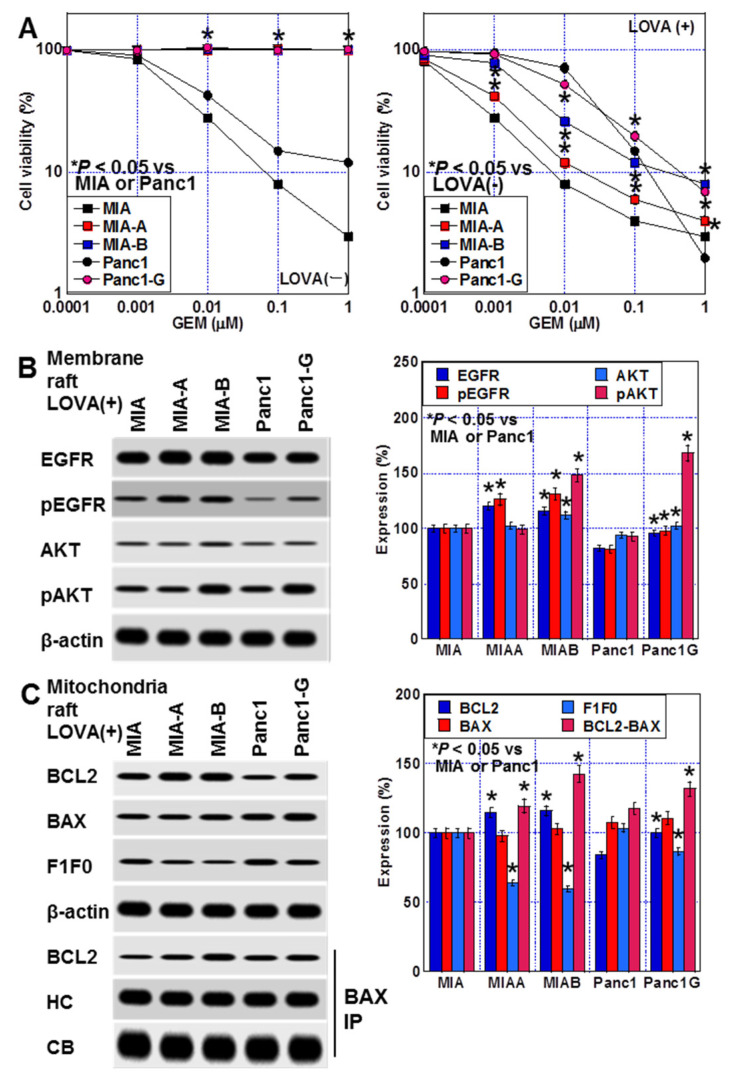
Effect of LOVA on GEM sensitivity in human GEM-resistant PDAC cells. (**A**) GEM sensitivity in GEM-resistant PDAC cell lines without LOVA (left) or with LOVA (right). (**B**) Effect of LOVA on EGFR and AKT activation in the plasma membrane raft fraction. (Right panel) Semiquantification of Western blot. (**C**) Effect of LOVA on mitochondrial raft proteins and BCL2-BAX binding in mitochondrial rafts. (Right panel) Semiquantification of Western blot. Error bars: standard deviation from three independent trials. Statistical significance was calculated using the Mann–Whitney U test. PDAC, pancreatic ductal adenocarcinoma; MIA, MIA-PaCa-2; C, control; LOVA, lovastatin; EGFR, epithelial growth factor receptor; pEGFR, phosphorylated EGFR; pAKT, phosphorylated AKT; PI3K, phosphoinositide 3-kinases; BCL2, B-cell lymphoma 2; BAX, Bcl-2-associated X protein; F1F0, F1F0 ATP synthase subunit C; GEM, gemcitabine.

When each resistant cell was treated with LOVA (0.2 μM, equivalent to IC = 20), the GEM sensitivity of MIA-A and Panc1-G in resistant cells increased compared with that in the parent cells. MIA-B also increased the GEM sensitivity to the same level as the parent cells (Figure 7A). LOVA treatment decreased the EGFR and AKT phosphorylation in the plasma membrane raft fraction in comparison with the LOVA(−) condition (Figure 7B). The binding between BCL2 and BAX in the mitochondrial raft fraction decreased in comparison with the LOVA(−) condition. The translocation of F1F0 ATP synthase subunit C to the mitochondrial raft was induced by LOVA treatment (Figure 7C).

## 3. Discussion

This study found that LOVA treatment inhibits the production of plasma membrane rafts and mitochondrial cholesterol and induces apoptosis in human PDAC cell lines. LOVA treatment also induced the occurrence of apoptosis in GEM-resistant PDAC cells.

LOVA treatment inhibits HMG-CoA reductase and suppresses mevalonate synthesis. As a result, the number of plasma membrane cholesterol rafts decreased in PDAC cells. The levels of proteins involved in the activation of cell survival signaling pathways, such as EGFR, PI3K, and AKT, were relatively high in plasma membrane rafts [26,27,28]. Our data showed that their activity was decreased and the mutual association was suppressed. As a result, the survival signals decreased, leading to the induction of apoptosis. Death receptors and downstream signaling molecules are recruited to these raft domains during apoptosis induction [17].

In contrast to plasma membrane rafts, studies related to mitochondrial rafts are limited. Mitochondrial cholesterol is mostly located in the membrane, with more in the outer membrane [30,31]. Cholesterol utilization is high in the inner membrane and low in the outer membrane [32,33]. Therefore, cholesterol rafts are thought to be present in the outer mitochondrial membrane. Lipid microdomains are also formed in the intracellular organelles, including the endoplasmic reticulum, Golgi apparatus, and the mitochondria, and are called raft-like microdomains [30]. Mitochondrial rafts are preferential sites for reactions related to the release of apoptosis-inducing factors [34].

In our data, cholesterol rafts were detected as well as plasma membranes, but the protein BCL2 contained therein was thought to bind to BAX, which inhibits apoptosis [35] By contrast, LOVA treatment inhibited the binding of these proteins and promoted apoptosis. Furthermore, the F1F0 ATP synthase subunit C, which is a candidate for the creation of mitochondrial permeability transition pores [36,37], migrates to the outer membrane rafts. This finding matches the reduced mitochondrial membrane potential associated with LOVA treatment, which may directly induce apoptosis.

According to the findings of our cell death inhibitor assay, the apoptosis inhibitors only caused a partial inhibition of LOVA-induced cell death. This observation suggests that cell death mechanisms other than apoptosis may be involved in LOVA-induced cell death. LOVA treatment increased the levels of superoxide, hydroxyl radicals, and hydrogen peroxide. Furthermore, the inhibition of oxidative stress by NAC partially inhibited cell death. These findings suggest that oxidative stress plays an important role in the induction of cell death by LOVA treatment. Oxidative stress triggers various types of cell death, including apoptosis, ferroptosis, necroptosis, and autophagy [38,39]. However, the inhibitors of necroptosis, ferroptosis, endoplasmic reticulum stress, and autophagy did not prevent the occurrence of LOVA-induced cell death. These results did not facilitate the identification of any specific type of cell death other than apoptosis. Therefore, future studies should further investigate the involvement of cell death other than apoptosis.

We have previously shown that mitochondrial targeting is useful for overcoming multidrug resistance [10,11]. GEM treatment damages the mitochondrial DNA and induces multidrug resistance by reprogramming energy metabolism and enhancing stemness. During mitochondrial alterations, the forced promotion of oxidative phosphorylation using medium-chain fatty acids is effective for overcoming multidrug resistance [11]. In addition to these energy alterations in drug-resistant cell lines, lipid rafts in the cell membrane and mitochondria are involved in anticancer drug resistance. Although previous studies reported that cholesterol rafts in the cell membrane are involved in GEM resistance [25], our data further demonstrated the importance of mitochondrial rafts. Therefore, mitochondria are important targets for overcoming anticancer drug resistance.

β-Hydroxylation by the liver is required for statin activation [19]. As shown in our experiment (Figure 1B), all statins, including LOVA, exhibit HMGCoA reductase inhibitory activity. They are then thought to be active forms. This suggests that cancer cells have the ability to make statins active. This knowledge may lead to the drug discovery of intratumoral metabolized statins which possess an antitumor effect. This is an issue that many researchers should tackle in the future.

The clinical significance of our study is that LOVA treatment effectively overcame GEM resistance in PDAC cell lines. Statins target the mitochondrial and plasma membrane rafts through their HMG-CoA reductase inhibitory action, which is their primary drug activity, to inhibit antiapoptotic resistance. Statins have been widely and safely used in the clinical setting, and clinical data related to their antitumor effects can be easily obtained. In the future, the early clinical application of statins as antitumor drugs is anticipated.

## 4. Materials and Methods

### 4.1. Cell Line and Reagents

MIA-PaCa-2 human PDA cell line was purchased from Dainihon Pharmaceutical Co. (Tokyo, Japan). PANC-1 cells were obtained from the American Type Culture Collection. The cells were cultured in Dulbecco’s Modified Eagle’s Medium supplemented with 10% fetal bovine serum at 37 °C in 5% CO_2_.

GEM-resistant MIA-A and MIA-B cells were established by continuous treatment with GEM (1 M; Sigma-Aldrich Inc., St. Louis, MO, USA) and CoCl_2_ (150 M; WAKO Pharmaceutical, Osaka, Japan) for >40 passages. GEM-resistant PANC-G cells were established by continuous treatment with GEM (1 M) for >40 passages [11].

### 4.2. Reagents

Statins (lovastatin, simvastatin, rosuvastatin, and pravastatin), GEM, N-acetyl-L-cysteine (NAC, 1 mM), chloroquine (10 μM), necrostatin (30 μM), cholesterol (2 mM) (Sigma), Z-VAD-FMK (ZVAD, 20 μM) (Santa Cruz Biotechnology, Santa Cruz, CA, USA), ferrostatin-1 (FRS, 2 μM) (Cayman Chemicals, Ann Arbor, MI, USA), and salubrinal (5 μM, Selleck, Houston, TX, USA) were purchased. All the statins and the other reagents were treated within 48 h.

### 4.3. Cell Growth, Cell Death, and Apoptosis

Cell growth was assessed using the 3-(4,5-dimethylthiazol-2-yl)-5-(3-carboxymethoxyphenyl)-2-(4-sulfophenyl)-2 H-tetrazolium (MTS) assay as previously described [10]. MTS assays were performed using a CellTiter 96 AQueous One Solution Cell Proliferation Assay kit (Promega Biosciences Inc., Madison, WI, USA). The plates were read using a Multiskan FC microplate photometer (Thermo Fisher Scientific, Tokyo, Japan) at a wavelength of 490 nm.

Cell death was assessed by pelleting floating and trypsinized adherent cells. The cell pellet was resuspended in 1 × PBS and 0.4% trypan blue (Sigma), and the cells were counted using a hemocytometer.

Apoptosis was induced by administering 0.5 μM lovastatine. DNA fragmentation was also observed. Genomic DNA was extracted using the TRIzol reagent (Thermo Fisher Scientific, Tokyo, Japan). Fragmented DNA was extracted from the supernatant by alcohol precipitation dissolved in Tris-EDTA buffer with a pH level of 8.0. DNA fragmentation was detected by gel electrophoresis, and the bands were stained with ethidium bromide for visualization under ultraviolet light. In addition, PARP cleavage due to apoptosis was examined by Western blotting.

### 4.4. Fluorescent Imaging

Mitochondrial function was examined using fluorescent probes, and 20 high-magnification fields were acquired using an all-in-one fluorescence microscope (KEYENCE, Osaka, Japan), with fluorescence intensity quantified on the same microscope. We used MitoROS (mitochondrial superoxide, SO) (10 μM, AAT Bioquest Inc., Sunnyvale, CA, USA), dihydrorhodamine 123 (mitochondrial hydrogen peroxide, HP) (10 μM, Sigma), and OxiORANGE (mitochondrial hydroxy radical, HR) (10 μM, Goryo Chemical, Sapporo, Japan) for assessing mitochondrial oxidative stress; MitoGreen (100 nM, PromoCell GmbH, Heidelberg, Germany) for assessing the mitochondrial volume, and tetramethylrhodamine ethyl ester (200 nM, Sigma) for mitochondrial membrane potential.

### 4.5. Chamber Invasion Assay

A modified Boyden chamber assay was performed to examine the in vitro invasive ability of the PDAC cells [40]. Following incubation at 37 °C for 24 h, the filters were carefully removed from the inserts, the cells were stained with hematoxylin for 10 min, and the stained cells were mounted on microscope slides. The number of stained cells in each insert was counted at 100× magnification. The invasive activity was quantified by calculating the average number of cells per well. All experiments were performed in triplicate.

Sphere assay with 1000 cells per well, which were seeded in 3D Tumorsphere Medium XF (Sigma) and cultured, was carried out. After 7 days, digital images of the spheres were acquired using a BZ-X710 all-in-one fluorescence microscope (KEYENCE, Osaka, Japan) and the size of the spheres was measured using NIH ImageJ software (version 1.52, Bethesda, MD, USA).

### 4.6. Protein Extraction

To prepare whole-cell lysates, cells were washed twice with cold PBS (Sigma) and harvested. Cells were lysed with RIPA buffer (Thermo Fisher Scientific) supplemented with 0.1% NP-40 [40]. Protein assays were performed using the Protein Assay Rapid Kit (WAKO).

### 4.7. Mitochondria Extraction

The cells were homogenized using a Teflon pestle homogenizer in extraction buffer (230 mM mannitol, 70 mM sucrose, 3 mM HEPES, and 0.1 mM EGTA; pH 7.4; 0.1% bovine serum albumin). The homogenate was centrifuged at 700× *g* for 10 min. The supernatant was decanted and centrifuged at 7000× *g* for 10 min. The pellet was resuspended in 40 mL of suspension buffer (230 mM mannitol, 70 mM sucrose, and 3 mM HEPES, pH 7.4) and re-centrifuged at 7000× *g* for 10 min. This step was repeated thrice. The final mitochondrial pellet was resuspended in 0.5 mL of suspension buffer.

### 4.8. Extraction of Raft Fractions

The lipid rafts were isolated from 5 × 10^7^ cells. Briefly, cell pellets disrupted in 1 mL of buffer A (50 mM Tris-HCl (pH 8.0), 10 mM MgCl2, 0.15 M NaCl, 1% Triton X-100, 5% glycerol, 50 mM PMSF, 1× protease inhibitor cocktail (Sigma), and 0.03% β-mercaptoethanol] were centrifuged for 5 min at 500× *g* and 4 °C. The obtained supernatant was added to 1 mL of buffer A (50 mM Tris-HCl (pH 8.0), 10 mM MgCl2, 0.15 M NaCl, and 80% sucrose) to achieve the final concentration of sucrose (40%). A discontinuous sucrose gradient was obtained by stratifying 7.5 mL of buffer A with 38% sucrose and 2 mL of buffer A with 15% sucrose. The gradient was ultracentrifuged for 18 h at 100,000× *g* and 4 °C. Twelve fractions (1 mL each) were collected from the top to the bottom of the gradient (F1–F12). Immunoblotting was performed to identify the fractions containing lipid rafts. Extraction of rafts from the mitochondrial fraction was performed in the same manner as above. To confirm that mitochondrial rafts were extracted, extracts (5 μg) were slot-blotted (Bio Rad, Tokyo, Japan) and stained with cholera toxin subunit B CF^®^Dye conjugates (10 μM, Biotium, Fremont, CA, USA).

### 4.9. Immunoblot Analysis

Lysates (20 μg) were subjected to immunoblot analysis by sodium dodecyl sulfate-polyacrylamide gel electrophoresis (12.5%) and then electrotransferred to nitrocellulose filters. The membrane was then incubated with the primary antibody followed by peroxidase-conjugated IgG antibody (Medical and Biological Laboratories, Nagoya, Japan). An anti-β-actin antibody was used to assess the level of protein loaded in each lane. Immune complexes were visualized using an enhanced chemiluminescence Western blot detection system (Amersham, Aylesbury, UK). The primary antibodies used in the study are shown in Table 1.

### 4.10. Immunoprecipitation

Immunoprecipitation was performed as previously described [41]. Briefly, whole-cell lysates were prewashed with lysis buffer containing protein A/G agarose (Santa Cruz) for 1 h at 4 °C and then centrifuged. The supernatant was incubated with antibody against BAX (Abcam) and protein A/G agarose for 3 h at 4 °C. The precipitate was collected by centrifugation, washed five times with lysis buffer, solubilized with sample buffer (Sigma, 40 μg), and subjected to immunoblot analysis.

### 4.11. Enzyme-Linked Immunosorbent Assay (ELISA)

Whole-cell lysates, membrane fractions, and mitochondrial fractions were prepared as previously described using RIPA buffer containing 0.1% SDS (Thermo Fisher Scientific) [41] and a mitochondria isolation kit for cultured cells (Thermo Fisher Scientific), respectively. Protein assays were performed using the Protein Assay Rapid Kit (WAKO). Using the extracted proteins, an ELISA kit was used to measure the cholesterol concentration (LabAssay Cholesterol kit, Fujifilm, Tokyo, Japan) and HMG-CoA reductase activity (HMG-CoA reductase assay kit, CS1090, Sigma) according to the manufacturer’s instructions.

### 4.12. Statistical Analysis

Statistical significance was calculated using a two-tailed analysis of variance test and unpaired Mann–Whitney tests using InStat software (version 3.1, GraphPad, Los Angeles, CA, USA). A two-sided *p*-value of <0.05 was considered significant.

## 5. Conclusions

In GEM-resistant cell lines, increased cell membrane rafts and mitochondrial rafts promote activation of EGFR and AKT and BCL2-BAX binding within the rafts, suppressing apoptosis, leading to GEM resistance. Lovastatin suppressed intracellular cholesterol production and reduced rafts, resulting in decreased GEM resistance. The results of our study may be useful in overcoming GEM resistance in PDAC and encouraging clinical approaches to achieve the antitumor effects of statins.

## Figures and Tables

**Table 1:** Antibodies.

Target	Clone	Dilution	Code	Manufacturer	Location
PARP	-	1:400	GTX100573	GeneTex	Irvine, CA, USA
Caveoin-1	A-6	1:300	sc-393013	Santa-Cruz	Santa Cruz, CA, USA
EGFR	CDX2-88	1:500	ab157524	Abcam	Waltham, MA, USA
pEGFR	PAb240	1:300	ab26	Abcam	Waltham, MA, USA
AKT	-	1:400	ab70346	Abcam	Waltham, MA, USA
pAKT	-	1:400	#9271	Cell Signaling ser473	Danvers, MA, USA
PI3K	OTI4H9	1:500	ab139307	Abcam	Waltham, MA, USA
BCL2	C-2	1:300	sc-7382	Santa-Cruz	Santa Cruz, CA, USA
BAX	E63	1:500	ab205822	Abcam	Waltham, MA, USA
F1F0	-	1:200	ab96655	Abcam	Waltham, MA, USA

PARP, poly (ADP-ribose) polymerase; EGFR, epithelial growth factor receptor; pEGFR, phosphorylated EGFR; pAKT, phosphorylated AKT; PI3K, phosphoinositide 3-kinases; BCL2, B-cell lymphoma 2; BAX, Bcl-2-associated X protein; F1F0, F1F0 ATP synthase subunit C.

## Data Availability

Data is contained within the article.

## Abbreviations

PDAC: pancreatic ductal carcinoma; GEM, gemcitabine; LOVA, lovastatin; MIA, MIA-PaCa-2; 5-FU, 5-fluorouracil; HMGC, 3-hydroxy-3-methylglutaryl coenzyme A; HMGCR, 3-hydroxy-3-methylglutaryl coenzyme A reductase; SIMVA, simvastatin; PRAVA, pravastatin; ROUSVA, rousvastatin; EGFR, epithelial growth factor receptor; PI3K, phosphatidylinositol-3 kinase; MβCD, methyl-β-cyclodextrin; BCL2, B-cell lymphoma 2; BAX, Bcl-2-associated X protein; PARP, poly (ADP-ribose) polymerase; NAC, N-acetyl-L-cysteine.
